# Intensive Inpatient vs. Home-Based Rehabilitation After Hip Fracture in the Elderly Population

**DOI:** 10.3389/fmed.2020.592693

**Published:** 2020-10-09

**Authors:** Yael Levi, Boris Punchik, Evgeniya Zikrin, David Shacham, Dori Katz, Evgeni Makulin, Tamar Freud, Yan Press

**Affiliations:** ^1^Faculty of Health Sciences, Joyce and Irving Goldman Medical School, Ben-Gurion University of the Negev, Beer-Sheva, Israel; ^2^Faculty of Health Sciences, Ben-Gurion University of the Negev, Beer-Sheva, Israel; ^3^Unit for Community Geriatrics, Division of Health in the Community, Ben-Gurion University of the Negev, Beer-Sheva, Israel; ^4^Home Care Unit, Clalit Health Services, Beer-Sheva, Israel; ^5^Siaal Research Center for Family Medicine and Primary Care, Faculty of Health Sciences, Ben-Gurion University of the Negev, Beer-Sheva, Israel; ^6^Department of Geriatrics, Soroka Medical Center, Beer-Sheva, Israel; ^7^Center for Multidisciplinary Research in Aging, Ben-Gurion University of the Negev, Beer-Sheva, Israel

**Keywords:** hip fracture, rehabilitation, home-based, inpatient, intensive

## Abstract

**Background:** As the population ages, the rate of hip fractures and the need for rehabilitation increases. Home-based rehabilitation (HBR) is an alternative to classic inpatient rehabilitation (IR), which is an expensive framework with non-negligible risks.

**Methods:** A retrospective study of patients 65 years and above following surgery to repair a hip fracture who underwent HBR or IR between 2016 and 2019. The two rehabilitation frameworks were compared for rehabilitation outcome and factors predicting successful rehabilitation. The outcome was determined with the Montebello Rehabilitation Factor Score-Revised (MRFS-R).

**Results:** Data were collected for 235 patients over 3 years. The mean age was 81.3 ± 8.0 and 172 (73.3%) were women. Of these, 138 underwent IR and 97 HBR. The HBR group had better family support and fewer lived alone. There were also differences in the type of fracture and surgery. The medical condition of the IR group was more complex, as reflected in a higher Charlson's comorbidity scores, higher rates for delirium and more infectious complications, a lower Norton score, lower serum hemoglobin, and albumin levels, and higher serum creatinine and urea levels. It also had a more significant functional decline after surgery and required a longer rehabilitation period. However, no difference was found in the rehabilitation outcomes between the two groups (MRFS-R ≥ 50). The independent predictors for rehabilitation in the IR group were serum albumin level, comorbidity, and cognitive state. There were no independent predictors in the HBR group.

**Conclusions:** In this retrospective study, there was no significant difference in short-term rehabilitation outcomes between the HBR and IR groups event though the patients in the IR group were medically more complex. This result should be taken into account when planning rehabilitation services after hip fracture and tailoring rehabilitation frameworks to patients.

## Introduction

Hip fracture, a common problem in elderly patient populations, is associated with functional impairment, reduced quality of life, and even mortality (1). The aim of rehabilitation by a multidisciplinary team is to improve the patient's functional condition and reduce negative health outcomes. The efficacy of rehabilitation processes has been assessed in multiple studies. In a latest systematic review of the literature and meta-analysis (2), the investigators found that compared to conventional care rehabilitation by a multidisciplinary team improves functional condition and motility. Previous studies reported a reduction in hospital day (3–5), a reduction in the risk of institutionalization following fracture (3, 5, 6), and economic efficiency (7). Over recent decades other models of home-based rehabilitation (HBR) have been developed in addition to the classical model. Two systematic reviews were published recently (8, 9) that evaluated the effectiveness of HBR for patients with fractures of the femoral neck, but most studies included in the review related to ongoing rehabilitation processes and not intensive rehabilitation immediately after surgery. Table 1 shows a summary of the main findings of six studies (10–15) that compared the effectiveness of HBR with that of standard inpatient rehabilitation (IR). In some of the studies, HBR was more effective than IR. However, in an analysis of administrative databases with a propensity-matched cohort of over 18,000 patients after a femoral neck fracture who were discharged to non-institutional rehabilitation in the community including HBR, the cost of community-based treatment including HBR was lower, but rehospitalization and mortality rates were higher (16).

**Table 1:** Home-based rehabilitation (HBR) vs. inpatient rehabilitation (IR): a review of studies.

**Study**	**Type**	**No of participants (HBR/IR)**	**Mean age (HBR/IR)**	**Women (%) (HBR/IR)**	**HBR intervention**	**Follow-up period**	**Outcomes: HBR better, than IR**	**Outcomes: IR better, than HBR**	**Outcomes: HBR same as IR**
(10)	Open-label. Only patients who gave consent entered HBR.	76/34	76.4/77.6	86/86	Max 12 days; N, PT-1.75 h, OT-2.37 h (accrued); Clinical responsibility-community GP	3 months	LoS in hospital; emotional distress		Physical mobility; pain; sleep; energy; social isolation, 90-days mortality rate; 90-days readmission rate
(11)	RCT	34/32	81.6/83.5	62/75	Mean 28.3 days; N, PT, OT, SW; Clinical responsibility-community GP	4 months	Functional status; Falls Efficacy Scale	Overall rehab time	4 months: Hospital readmission; career time; visits to GPs; use of community services; falls; Berg Balance score; Satisfaction Total Scale; Caregiver Strain Index
(12)	RCT	41/40	75 for all	60% for all	Mean 4.6 visits of PT and 1.5 visits of N/patient/all period; Clinical responsibility-NA	1 year	Scores for community and household ambulation; rehab time		
(13)	Open-label. Patients who gave consent to HBR were randomized to IR/HBR groups	95/99	83.6 for all	85.5% for all	NA	1 year	Functional status		
(14)	RCT	107/98	83.2/82.6	73.8/69.4	N, PT, OT, SW, dietician; Clinical responsibility- geriatrician	1 year	LoS in geriatric ward		1-year mortality; indoor and outdoor walking ability; indoor and outdoor walking device use
(15)	Open-label. Only patients who gave consent entered HBR	44/134	NA	NA	N, PT, OT; Clinical responsibility-community GP	Only rehab period (average 49.4 days for HBR and 61.1 days for IR)	Acute hospital LoS; rehabilitation time; total cost		Functional gain/loss ratio (Heinemann index)

*HBR, home-based rehabilitation; IR, inpatient rehabilitation; PT, Physiotherapist; N, nurse; OT, Occupational Therapist; SW, social worker; GP, general practitioner; LoS, length of stay; NA- non-available*.

In recent years, the Clalit Healthcare Services, the largest in Israel, developed an HBR program in its Southern District. The aim of the present study was to compare patients who had surgery for femoral neck fractures and underwent HBR with those who were rehabilitated in the Geriatrics Department of a tertiary university hospital with IR. There were three primary study questions:

1. Is there a difference between patients who underwent rehabilitation in these two frameworks in a real-life setting?
2. Is there a difference in rehabilitation outcome between the two frameworks?
3. Are there different predictors of success in the two rehabilitation settings?

## Methods

### Setting

In the Southern District of the Clalit Healthcare Services recently established a Geriatric Sector including a Geriatrics department in the tertiary Soroka University Medical Center, and a community Geriatrics Service with an HBR unit.

#### Geriatrics Department

The Soroka University Medical Center is a 1,150-bed tertiary hospital, the biggest in southern Israel and the third largest in Israel. The hospital has a 25-bed Geriatrics ward that was established over 30 years ago. The staff includes specialists and residents in geriatric medicine, nurses and aides, physiotherapists, an occupational therapist, a dietician, and a social worker. It provides consultation services for the medical center's wards and units. The ward admits patients with acute illnesses referred from the emergency room and patients in need of rehabilitation, some following surgical repair of a femoral neck fracture. The process for admitting these patients begins with a geriatric consultation in the two Orthopedic Surgery wards of the hospital over the 1st days following surgery, including an initial geriatric assessment. Patients who meet criteria for HBR are given this option. Those who do not meet the criteria are referred for IR. Based on criteria including available space in the Geriatrics ward, health insurance from an HMO that does not support rehabilitation in the Geriatrics ward, the patient's or the family's decision to undergo rehabilitation elsewhere than the Soroka Medical Center, and the anticipated need for a prolonged rehabilitation due to a very poor functional or cognitive state, the patient is transferred either to the Geriatrics ward in the medical center or to another rehabilitation center outside of the hospital. Over the course of their stay in the Geriatrics ward each patient undergoes a comprehensive geriatric assessment. All healthcare professionals in the unit take part in this assessment, with an emphasis on medical problems, drug therapy, the patient's cognitive and affective state, and the patient's functional and social condition, including support resources and living conditions. In accordance with the assessment findings an interventional rehabilitation plan is developed.

This plan is adjusted during the course of the day's work and at weekly meetings of the multidisciplinary staff. The rehabilitation process includes mobilization facilitated by all staff members, physiotherapy five times per week (average 45 min per session), occupational therapy several times a week (average 30 min per session), and a psychosocial intervention by the social worker. Preparation for discharge from the ward begins at the early stages of hospitalization. It includes interaction with relevant community services to adjust living conditions, nursing help, and ongoing rehabilitation at home. Patients with femoral neck fracture are discharged when the goals of rehabilitation are achieved such as independence using accessories in passageways, and mobility and independence in toileting, or when the patient reaches a plateau based on the Functional Independence Measure (FIM) (17).

#### The Unit for HBR

This unit includes a multidisciplinary staff with geriatric specialists, nurses, occupational therapists, physiotherapists, a dietician, and a social worker. This is a nuclear staff with tenured positions and do not work for extra income. Weekly staff meetings are held. The rehabilitation program begins with the receipt of patient data from the Orthopedic Surgery wards, an additional evaluation using criteria for HBR, and determination of the discharge date. At the time of discharge from the hospital the staff conducts a house visit including a comprehensive geriatric assessment, and determines the goals of the rehabilitation program, similar to the preparations made at the Geriatrics ward in the hospital. Over the course of rehabilitation the patient lives in the framework of home hospitalization under the responsibility of a geriatric specialist and receives comprehension care, including 24-h availability of the rehabilitation staff's doctor and nurse. The patient has a 45-min physiotherapy session 5 days a week, occupational therapy at least once a week (average 30 min per session), and psychosocial intervention by the staff social worker, as needed. In addition, the Clalit Healthcare Services pays for required nursing care for the patient during HBR.

The decision to discharge from HBR is reached using the same criteria as in the Geriatric ward, when the goals of rehabilitation are achieved or the patient reaches a plateau based on FIM. In most cases, the patient then continues rehabilitation at home or in a rehabilitation center, but at a lower level of intensity.

It is important to note that the staffs of the unit for intensive rehabilitation and of the Geriatric ward are in constant contact including convening weekly joint staff meetings. All the geriatric specialists in both frameworks undergo their training in geriatric medicine in the Geriatrics ward of the Soroka Medical Center.

Criteria for admission to HBR. The unit for home-based rehabilitation adopted the criteria of Crotty et al. (18) for admission to the unit:

1. Rehabilitation is indicated;
2. Discharge from the hospital is appropriate;
3. The patient's physical and cognitive condition enable active participation in a rehabilitation program;
4. The home environment is suitable for home rehabilitation;
5. There is a healthcare provider in the community who can provide for the medical needs of the patient, in this case the geriatrician on the HBR staff is the responsible medical provider;
6. The main caregiver, if there is one, agrees with the decision to apply home-based rehabilitation.

### Study Population

The study population consisted of patients 65 years or above who underwent surgical repair of a femoral neck fracture and were admitted for rehabilitation in the Geriatrics ward of the Soroka University Medical Center (inpatient rehabilitation, IR) or with the Unit for Home-based Rehabilitation (HBR) of the Clalit Healthcare Services between January 1, 2016 and December 31, 2019.

### Data Sources

Data on the study participants were obtained retrospectively from the electronic medical records (EMR) of the Soroka University Medical Center and the Clalit Healthcare Services. The following data were collected:

1. Socio-demographic data—age, gender, education level, marital status, place of residence, having a staircase or elevator at home, information about the patient's main caregiver, nursing caregiver;
2. Medical history—type of hip fracture, type of surgery, the patient's chronic diseases [the Charlson comorbidity index (19) score was calculated], information about complications during rehabilitation (delirium, thromboembolism, pressure sores, cardio-vascular problems, and infections), drug therapy and laboratory tests on admission to rehabilitation site;
3. Length of stay (LoS) at the orthopedic and geriatric wards or at the HBR unit, emergency room referrals and hospitalizations during rehabilitation (HBR group only) and six months after the rehabilitation process, and mortality rate;
4. Functional status as assessed by the Functional Independence Measure—anamnestic FIM (anFIM), FIM on admission (FIMa), and FIM on discharge (FIMd). FIM is an 18-item ordinal scale with scores on each item ranging from 1 to 7 (1 = total assistance and 7 = complete independence. Scores below 6 require supervision or assistance) with the total score ranges from 18 to 126;
5. Cognitive function was assessed with the Mini Mental State Examination (MMSE) (20), with the global score ranging from 0 to 30 where a higher score indicates a better cognitive status;
6. Mood status was assessed by geriatricians during CGA and a diagnosis of depression was made based on the DSM 5 criteria (21);
7. The risk for pressure sores was assessed by the Norton screening tool for pressure ulcer risk, rated on a scale of 0–20 with higher scores indicating a lower probability for pressure ulcers (22);
8. Rehabilitation outcome was measured with the Montebello Rehabilitation Factor Score Revised (MRFS-R) (23). The MRFS-R is based on the differences between an FIM, FIMa, and FIMd, calculated according to the following formula:

MRFS-R = ((FIMd – FIMa)/FIMd)/((an FIM – FIMa)/an FIM) × 100

This index enables an appraisal of the degree that patients realized their rehabilitation potential. For example, a MRFS-R score of 55 indicates that the patient achieved 55% of the rehabilitation potential.

### Statistical Analysis

Data analysis was performed using the IBM SPSS Statistics Version 26 software. Univariate data analysis was performed to assess the relationship between the degree of functional improvement and rehabilitation in the IR and HBR groups and in the entire study population. For normally distributed continuous quantitative variables, the Student's *T*-Test or One-Way ANOVA were used. For continuous variables that were not normally distributed the Mann–Whitney or Kruskal–Wallis test were used. For quality variables the Chi-Square test was used.

The multivariate analysis included the following variables: (a) those with a statistically significant difference in the univariate analysis (*p* < 0.05), and (b) those with clinical significance (e.g., gender). Before performing the model, we checked for interactions or confounders and removed them from the model. A logistic regression model was built for each type of rehabilitation to predict its success, which was defined as MRFS-R ≥ 50% for the study. We added a logistic regression model to predict the success of rehabilitation for the entire study population with the addition of the independent variable “Rehabilitation setting” (HBR vs. IR).

A two-sided *p* < 0.05 was considered statistically significant for all tests. The study was approved by the Helsinki Committee of the Soroka Medical Center (SOR-0211-19).

## Results

Data were collected for 235 patients with a mean age of 81.3 ± 8.0, of who 172 (73.3%) were women. The IR group included 138 patients and the HBR group 97.

### Baseline Characteristics of the IR and HBR Groups (Table 2)

The IR group had fewer married patients (56.7 vs. 33.3%, *P* = 0.001), more patients who lived alone (41.3 vs. 10.0%, *P* < 0.001), and fewer patients with a partner who played the role of main caregiver (41.2 vs. 32.6%, *P* = 0.008). The IR group had fewer patients with an intracapsular fracture (30.4 vs. 48.5%, *P* = 0.008), and there was a corresponding difference in the type of surgery: in the patients who had an intrascapular fracture, 51.2% in the IR group had total hip replacement (THR) while 88.1% of the patients in the HBR group did (*P* < 0.001). The IR group had more complex medical issues with a higher total score in the Charlson's Comorbidity Index (TCS) (5.9 ± 1.9 vs. 5.2 ± 2.4, *P*=0.001), they were taking more chronic mediations at admission to rehabilitation (10.2 ± 1.9 vs. 7.5 ± 2.9, *P* < 0.001), their Norton score was lower (13.6 ± 1.5 vs. 14.9 ± 2.8, *P* < 0.001), the serum hemoglobin level was decreased more after surgery (2.5 ± 1.5 vs. 2.0 ± 1.1, *P* = 0.001), the admission serum creatine level was higher (1.4 ± 1.5 vs. 1.1 ± 0.6, *P* = 0.016) as was the admission serum urea level (79.9 ± 37.8 vs. 56.5 ± 27.0, *P* < 0.001), and the serum albumin level was lower (3.0 ± 0.4 vs. 3.2 ± 0.5, *P* = 0.004). Although there were no statistically significant differences between the two rehabilitation groups in anFIM, the FIMa was lower in the IR group (65.1 ± 11.4 vs. 76.8 ± 26.9, *P* < 0.001) and the FIM loss (anFIM-FIMa) following the femur fracture in this group was more prominent (46.2 ± 13.0 vs. 27.7 ± 15.3, *P* < 0.001).

**Table 2:** Baseline characteristics of the IR and HBR groups.

	**IR (*N* = 138)**	**HBR (*N* = 97)**	***P*-value**
Age (years), mean ± SD	82.5 ± 7.10	80.2 ± 8.9	0.084
Gender (female), *n* (%)	107 (77.5)	65 (77.0)	0.101
Family status (married), *n* (%)	46 (33.3)	55 (56.7)	0.001
Living status (alone), *n* (%)	57 (41.3)	10 (10.3)	<0.001
Nursing caregiver (yes), *n* (%)	127 (92.0)	94 (96.9)	0.127
Main caregiver (spouse), *n* (%)	29 (32.6)	36 (41.2)	0.008
**Home accessibility**, ***n*** **(%)**			
No stairs, yes elevator	66 (47.8)	51 (52.6)	0.592
Yes stairs, no elevator	63 (45.7)	38 (39.2)	
Missing data	9 (6.5)	8 (8.3)	
**Education**, ***n*** **(%)**	Choose		
≤ 4 years	43 (31.2)	40 (41.2)	0.114
5–8 years	17 (12.3)	17 (17.5)	
9–12 years	31 (22.5)	19 (19.6)	
>12 years	47 (34.1)	21 (21.5)	
**Fracture type (intracapsular)**, ***n*** **(%)**	42 (30.4)	47 (48.5)	0.008
**Surgery Type**, ***n*** **(%)**			
Internal fixation	95 (68.8)	55 (56.7)	
Hemiarthroplasty	22 (15.9)	5 (5.2)	<0.001
Total hip replacement	21 (15.2)	37 (38.1)	
LoS Orthopedics, days, mean ± SD	7.0 ± 2.9	7.8 ± 9.8	0.155
**Medical status**			
TCS, mean ± SD	5.9 ± 1.9	5.2 ± 2.4	0.001
Medication number, mean ± SD	10.2 ± 3.3	7.5 ± 2.9	<0.001
MMSE, mean ± SD	21.5 ± 6.3	20.0 ± 8.9	0.689
Depression, *n* (%)	23 (16.7)	18 (18.6)	0.836
Norton, mean ± SD	13.6 ± 1.5	14.9 ± 2.8	<0.001
BMI, mean ± SD	24.7 ± 5.2	25.7 ± 5.0	0.175
**Laboratory**			
Hemoglobin before surgery (g/dl), mean ± SD	12.1 ± 2.0	12.4 ± 1.54	0.223
Hemoglobin after surgery (g/dl), mean ± SD	9.5 ± 1.8	10.4 ± 1.6	0.002
Loss of Hemoglobin (g/dl), mean ± SD	2.5 ± 1.5	2.0 ± 1.1	0.001
Serum creatinine (mg/dl), mean ± SD	1.4 ± 1.5	1.1 ± 0.6	0.016
Serum urea (mg/dl), mean ± SD	72.9 ± 37.8	56.5 ± 27.0	<0.001
Serum sodium (mEq/L), mean ± SD	135.6 ± 4.5	135.6 ± 4.4	0.863
Serum albumin (g/dl), mean ± SD	3.0 ± 0.4	3.2 ± 0.5	0.004
**Functional status**			
anFIM, mean ± SD	111.3 ± 15.0	104.5 ± 28.2	0.775
FIM a, mean ± SD	65.1 ± 14.1	76.8 ± 26.9	<0.001
FIM loss (anFIM-FIMa)	46.2 ± 13.0	27.7 ± 15.3	<0.001

*HBR, home-based rehabilitation; IR, inpatient rehabilitation; TCS, the Charlson Co-morbidity Index, total score; MMSE, the Mini Mental State Examination; BMI, body mass index; anFIM, anamnestic Functional Independence Measure; FIMa, Functional Independence Measure on admission; FIMd, Functional Independence Measure on discharge*.

### The Treatment Routine

Over the course of rehabilitation in the HBR group the patients underwent a mean of 18.6 ± 5.6 visits by different staff members, with 1.6 ± 0.8 by a geriatrics specialist, 2.5 ± 0.9 by a nurse, 10.5 ± 3.4 by a physiotherapist, 2.2 ± 2.1 by an occupational therapist, 0.4 ± 0.6 by a dietician, and 1.3 ± 0.7 by a social worker. The patients in the IR group were treated in line with the work routine described above, contact with the medical and nursing team was available at all times, daily treatment by the physiotherapist and occupational therapist, multiple contacts with the social worker, and at least one consultation with a dietician.

### Complications During Rehabilitation, use of Healthcare Services, and Mortality Rates (Table 3)

Patients in the IR group had a higher occurrence of delirium (21.7 vs. 9.3%, *P* = 0.016) and infectious diseases (29.0 vs. 9.3%, *P* < 0.001). However, patients in the HBR group had more pressure sores (12.4 vs. 2.9%, *P* = 0.007).

**Table 3:** Morbidity during rehabilitation, rehabilitation outcome, medical system utilization and mortality rate in the IR and HBR groups.

	**IR (*N* = 138)**	**HBR (*N* = 97)**	***P*-value**
**Morbidity during rehab process**, ***n*** **(%)**			
Delirium	30 (21.7)	9 (9.3)	0.016
Any infection	40 (29.0)	9 (9.3)	<0.001
Cardiovascular (IHD, CHF, stroke)	33 (23.9)	23 (23.7)	>0.999
Periprosthetic Fracture	5 (3.6)	1 (1.0)	0.405
Thromboembolism	3 (2.2)	0 (0)	0.270
Pressure Ulcers	4 (2.9)	12 (12.4)	0.007
**Outcomes**			
FIM discharge, mean ± SD	83.6 ± 19.0	91.3 ± 29.1	<0.001
FIM gain (FIMd-FIMa), mean ± SD	18.5 ± 13.3	14.6 ± 11.0	0.002
MRFS-R, mean ± SD	47.9 ± 38.4	51.5 ± 28.3	0.950
Patients with MRFS-R ≥ 50, *n* (%)	89 (64.5)	57 (58.8)	0.377
**Medical system utilization**, ***n*** **(%)**			
Rehabilitation time (days), mean ± SD	20.9 ± 7.5	15.6 ± 4.0	<0.001
At least 1 ER visit/6 months, *n* (%)	21 (15.2)	32 (33.0)	0.002
At least 1 hospitalization/6 months, *n* (%)	26 (18.8)	20 (20.6)	0.860
**Mortality**, ***n*** **(%)**			
During rehab	2 (1.4)	0 (0)	0.513
Within 90 days after rehab	11 (8.0)	6 (6.2)	0.799

*HBR, home-based rehabilitation; IR, inpatient rehabilitation; IHD, ischemic heart disease; CHF, congestive heart failure; FIMa, Functional Independence Measure on admission; FIMd, Functional Independence Measure on discharge; MRFS-R, the Montebello Rehabilitation Factor Score-Revised; ER, emergency room*.

The period of rehabilitation was longer in the IR group (20.9 ± 7.5 days vs. 15.6 ± 4.0, *P* < 0.001). Over the 6 months following the end of the rehabilitation program 33% of the patients in the HBR group went to the emergency room at least once compared to 15.2% in the IR group (*P* = 0.002). There was no statistically significant difference between the groups in hospitalization over this follow-up period. Two patients in the IR group died during rehabilitation while all the patients in the HBR group survived the rehabilitation period (*P* = 0.513). There was no statistically significant differences between the groups in the three-month mortality rate (8.0 vs. 6.2%, *P* = 0.799).

### Outcome of Rehabilitation

At the end of the rehabilitation process the FIMd score was higher in the HBR group (91.3 ± 29.0 vs. 83.6 ± 19.9, *P* < 0.001), but the FIM gain (FIMd – FIMa) was lower (14.6 ± 11.0 vs. 18.5 ± 13.3, *P* = 0.002). There were no statistically significant differences between the groups in the MRSF-R score (51.5 ± 28.3 vs.47.9 ± 38.4, *P* = 0.95) and there was no difference in the number of patients that had a score of MRFS-R ≥ 50. In the total study population of 235 patients in the two rehabilitation settings, 146 (62.1%) achieved a score of MRFS-R ≥ 50 (Table 4). In a univariate analysis we found that younger age, a lower TCS, higher MMSE and Norton scores, a higher serum albumin level, and the absence of a periprosthetic fracture during the course of rehabilitation were associated with a successful rehabilitation. A multivariate logistic regression model was developed with all the variables that had a *P* < 0.1, sex, age, and rehabilitation setting (HBR vs. IR). Only two independent variables were associated with rehabilitation success, TCS (OR = 0.86, 95% CI = 0.74–0.99) and MMSE (OR = 1.07, 95% CI = 1.01–1.13 (*P* = 0.014). Thus, in the multivariate analysis the rehabilitation setting was not associated with rehabilitation success.

**Table 4:** Baseline characteristics of all participants (*N* = 235) according to rehabilitation outcomes (MRFS-R <50 vs. MRFS-R ≥ 50).

	**MRFS-R <50 (*N* = 89)**	**MRFS-R ≥ 50 (*N* = 146)**	***P*-value**
Age (years), mean ± SD	83.2 ± 8.1	80.5 ± 7.7	0.013
Gender (female), *n* (%)	63 (70.8)	103 (70.5)	1.000
Family status (married), *n* (%)	35 (39.3)	66 (45.2)	0.416
Living status (alone), *n* (%)	22 (24.7)	45 (30.8)	0.372
Nursing caregiver (yes), *n* (%)	81 (92.0)	139 (95.2)	0.127
Main caregiver (spouse), *n* (%)	29 (32.6)	36 (41.2)	0.396
**Education**, ***n*** **(%)**			
≤ 4 years	32 (36.0)	51 (34.9)	0.274
5–8 years	8 (9.0)	26 (17.8)	
9–12 years	22 (24.7)	28 (19.2)	
>12 years	27 (30.3)	41 (28.1)	
Fracture type (intracapsular), *n* (%)	32 (36.0)	57 (39.0)	0.679
**Surgery Type**, ***n*** **(%)**			
Internal fixation	59 (66.3)	55 (56.7)	<0.0001
Hemiarthroplasty	12 (13.5)	5 (5.2)	
Total hip replacement	18 (20.2)	37 (38.1)	
LoS Orthopedics, days, mean ± SD	7.4 ± 3.2	7.3 ± 8.1	0.907
**Medical status**			
TCS, mean ± SD	6.2 ± 2.2	5.3 ± 2.1	0.001
Medication number, mean ± SD	9.1 ± 3.1	9.1 ± 3.5	0.904
MMSE, mean ± SD	18.3 ± 8.8	22.4 ± 6.2	<0.0001
Depression, *n* (%)	16 (18.0)	25 (17.1)	0.861
Norton, mean ± SD	13.5 ± 2.4	14.6 ± 2.1	<0.0001
BMI, mean ± SD	24.4 ± 5.9	25.7 ± 4.5	0.126
**Laboratory**			
Loss of Hemoglobin (g/dl), mean ± SD	2.4 ± 1.3	2.3 ± 1.4	0.469
Serum creatinine (mg/dl), mean ± SD	1.3 ± 1.2	1.3 ± 1.2	0.795
Serum urea (mg/dl), mean ± SD	70.5 ± 31.8	63.5 ± 36.1	0.134
Serum sodium (mEq/L), mean ± SD	135.4 ± 3.8	135.4 ± 4.8	0.381
Serum albumin (g/dl), mean ± SD	3.0 ± 0.4	3.2 ± 0.4	0.022
**Functional status**			
anFIM, mean ± SD	102.7 ± 25.6	112.0 ± 18.1	0.003
FIM a, mean ± SD	68.2 ± 23.2	71.0 ± 19.0	0.327
FIM loss (anFIM-FIMa)	34.5 ± 18.5	41.1 ± 14.9	0.003
**Morbidity during rehab process**, ***n*** **(%)**			
Delirium	20 (22.5)	19 (13.0)	0.071
Any infection	22 (24.7)	27 (18.5)	0.321
Cardiovascular (IHD, CHF, stroke)	20 (22.5)	36 (24.7)	0.756
Periprosthetic Fracture	5 (5.6)	1 (0.7)	0.030
Thromboembolism	1 (1.1)	2 (1.4)	1.000
Pressure Ulcers	10 (11.2)	6 (4.1)	0.058
**Rehabilitation time (days), mean** ± **SD**	18.9 ± 7.5	19.4 ± 7.8	0.601
**Place of rehabilitation**			
Home-based rehabilitation	40 (40.9)	57 (39.0)	0.413
Inpatient rehabilitation	49 (55.1)	89 (61.0)	

*TCS, the Charlson Co-morbidity Index, total score; MMSE, the Mini Mental State Examination; BMI, body mass index; anFIM, anamnestic Functional Independence Measure; FIMa, Functional Independence Measure on admission*.

### Characteristics of Patients With MRFS-R ≥ 50 in the IR and HBR Groups

Table 5 shows the results of univariate comparisons of socio-demographic and health variables and length of stay in the orthopedic surgery and rehabilitation wards between patients who had MRFS-R ≥ 50 and those with MRFS-R <50 in each of the study groups. The patients in the IR group who achieved this score were younger (81.5 ± 7.0 vs. 84.3 ± 7.1, *P* = 0.031), with lower co-morbidity (TCS, 5.6 ± 1.7 vs. 6.5 ± 2.0, *P* = 0.008), a higher MMSE score (22.5 ± 5.8 vs. 19.4 ± 6.9, *P* = 0.008), a higher Norton score (13.9 ± 1.5 vs. 13.1 ± 1.5, *P* = 0.004), a higher serum albumin level (3.1 ± 0.4 vs. 2.9 ± 0.3, *P* = 0.007), a lower percentage of delirium (15.7 vs. 32.7%, *P* = 0.03), and fewer cases of periprosthetic fractures (0 vs. 10.2%, *P* = 0.005). The length of stay (LoS) in the orthopedic surgery ward prior to rehabilitation in the IR group was shorter among patients with MRFS-R ≥ 50, but this difference did not reach statistical significance (6.7 ± 2.7 vs. 7.7 ± 3.2 days, *P* = 0.06). The picture was a little different in the HBR group. In patients with MRFS-R ≥ 50 the TCS score was lower (4.8 ± 2.4 vs. 5.9 ± 2.4, *P* = 0.03), the MMSE was higher (22.3 ± 6.9 vs. 17.1 ± 10.4, *P* = 0.008), and the Norton score was higher (15.6 ± 2.4 vs. 14.0 ± 3.1, *P* = 0.008). At admission to rehabilitation their serum urea level was lower (40.9 ± 24.3 vs. 64.4 ± 28.8, *P* = 0.018). Two other variables that showed a trend to difference that did not reach statistical significance were a higher percentage of patients who underwent THR compared to other types of surgery (45.6 vs. 27.5%, *P* = 0.075) and a longer LoS in rehabilitation in the MRFS-R ≥ 50 group (17.3 ± 8.0 vs. 15.1 ± 4.2 days, *P* = 0.078).

**Table 5:** Predictors of MRFS-R ≥ 50.

	**Inpatient Rehabilitation**			**Home-based rehabilitation**		
	**MRFS-R <50**	**MRFS-R ≥ 50**	***P***	**MRFS-R <50**	**MRFS-R ≥ 50**	***P***
Age (years), mean ± SD	84.3 ± 7.1	81.5 ± 7.0	0.031	81.9 ± 9.1	79.0 ± 8.6	0.116
Gender (female), *n* (%)	36 (73.5)	65 (73.0)	1.000	27 (67.5)	38 (66.7)	1.000
Family status (married), *n* (%)	15 (30.6)	31 (34.8)	0.707	20 (50.0)	22 (38.6)	0.302
Living status (alone), *n* (%)	20 (40.8)	37 (41.6)	1.000	2 (5.0)	8 (14.0)	0.189
Nursing caregiver (yes), *n* (%)	42 (85.7)	84 (94.4)	0.104	39 (97.5)	55 (96.5)	1.000
Main caregiver (spouse), *n* (%)	10 (20.4)	19 (21.3)	0.980	11 (27.5)	25 (43.9)	0.107
Fracture type (intracapsular), *n* (%)	14 (28.6)	28 (31.5)	0.847	18 (45.0)	29 (50.9)	0.680
Surgery type (THR), *n* (%)	7 (14.3)	14 (15.7)	0.839	11 (27.5)	26 (45.6)	0.075
LoS Orthopedics (days), mean ± SD	7.7 ± 3.2	6.7 ± 2.7	0.060	7.1 ± 3.3	8.3 ± 12.6	0.397
**Medical status**						
(TCS), mean ± SD	6.5 ± 2.0	5.6 ± 1.7	0.008	5.9 ± 2.4	4.8 ± 2.4	0.03
Medication number, mean ± SD	10.1 ± 3.2	10.3 ± 3.3	0.749	8.0 ± 2.6	7.2 ± 3.1	0.209
MMSE, mean ± SD	19.4 ± 6.9	22.5 ± 5.8	0.008	17.1 ± 10.4	22.3 ± 6.9	0.008
Norton, mean ± SD	13.1 ± 1.5	13.9 ± 1.5	0.004	14.0 ± 3.1	15.6 ± 2.4	0.008
BMI, mean ± SD	24.1 ± 6.5	25.0 ± 4.2	0.459	24.8 ± 5.3	26.4 ± 4.7	0.148
**Laboratory**						
Loss of hemoglobin (g/dl), mean ± SD	2.6 ± 1.4	2.5 ± 1.6	0.561	2.1 ± 1.1	1.9 ± 1.0	0.416
Serum creatinine (mg/dl), mean ± SD	1.4 ± 1.5	1.4 ± 1.5	0.912	1.2 ± 0.7	1.0 ± 0.6	0.156
Serum urea (mg/dl), mean ± SD	75.4 ± 33.6	71.5 ± 40.1	0.564	64.4 ± 28.8	40.9 ± 24.3	0.018
Serum albumin (g/dl), mean ± SD	2.9 ± 0.3	3.1 ± 0.4	0.007	3.2 ± 0.4	3.3 ± 0.5	0.296
**Morbidity during rehabilitation**						
Depression, *n* (%)	6 (12.2)	17 (19.1)	0.348	10 (25.0)	8 (14.0)	0.193
Delirium, *n* (%)	16 (32.7)	14 (15.7)	0.030	4 (10.0)	5 (8.8)	1.000
Any infection, *n* (%)	15 (30.6)	19 (21.3)	0.302	6 (15.0)	3 (5.3)	0.155
Cardiovascular (IHD, CHF, stroke), *n* (%)	3 (6.1)	6 (6.7)	1.000	2 (5.0)	2 (3.5)	1.000
Periprosthetic Fracture, *n* (%)	5 (10.2)	0 (0.0)	0.005	0 (0.0)	1 (1.8)	1.000
Thromboembolism, *n* (%)	1 (2.0)	2 (2.2)	1	0	0	
Pressure Ulcers, *n* (%)	3 (6.1)	1 (1.1)	0.224	7 (17.5)	5 (8.8)	0.224
Rehabilitation time (days), mean ± SD	22.1 ± 8.1	20.8 ± 7.4	0.373	15.1 ± 4.2	17.3 ± 8.0	0.078

*MRFS-R, the Montebello Rehabilitation Factor Score-Revised; THR, total hip replacement; LoS, length of stay; TCS, the Charlson Co-morbidity Index; MMSE, the Mini Mental State Examination; BMI, body mass index; IHD, ischemic heart disease; CHF, congestive heart failure*.

### Logistic Regression Analysis Predicting MRFS-R ≥ 50

Separate regression models were built for the two study groups. The models included sex and variables that reached statistical significance in univariate analyses or had a *P*-value lower than 0.1 for MRFS-R ≥ 50 (Table 5). The model for IR is shown in Table 6. In the final model, with all the relevant variables only, TCS (OR = 0.73, 95% CI = 0.58–0.93, *P* = 0.01), MMSE (OR = 1.09, 95% CI = 1.01–1.16, *P* = 0.018) and serum albumin level prior to the start of rehabilitation (OR = 5.97, 95% CI = 1.65 = 21.66, *P* = 0.007) predicted a successful rehabilitation outcome with MRFS-R ≥ 50.

**Table 6:** Hierarchical logistic regression analyses predicting MRFS-R≥50 in IR patients.

**Variables**	**Model 1**	**Model 2**	**Model 3**	**Final model**
	**OR (95% CI)**, ***P***	**OR (95% CI)**, ***P***	**OR (95% CI)**, ***P***	**OR (95% CI)**, ***P***
Gender (male)	0.91 (0.34–2.5), 0.851	0.82 (0.30–2.27),0.702	0.91 (0.34–2.45), 0.851	0.93 (0.35–2.25), 0.878
TCS	0.75 (0.59–0.95), 0.016	0.76 (0.60–0.96), 0.022	0.73 (0.58–0.93), 0.010	0.73 (0.58–0.93), 0.010
MMSE	1.09 (1.01–1.18), 0.023	1.08 (0.99–1.17), 0.063	1.08 (1.01–1.16), 0.021	1.09 (1.01–1.16), 0.018
Albumin	5.56 (1.51–20.44), 0.010	5.23 (1.34–20.36), 0.017	6.68 (1.75–22.46), 0.005	5.97 (1.65–21.66), 0.007
Norton	1.08 (0.78–1.50), 0.651	–	–	–
Delirium		0.71 (0.22–2.35), 0.577	–	–
Periprosthetic fracture		0.00 (0.00–0.00), 0.999	–	–
Rehabilitation time			1.0 (0.9–1.0), 0.502	–

MRFS-R, the Montebello Rehabilitation Factor Score-Revised, IR, inpatient rehabilitation; TCS, the Charlson Comorbidity Index, total score; MMSE, the Mini Mental State Examination.

Model 1: Socio-demographic and clinical variables before rehabilitation.

Model 2: With the addition of data collected over the course of rehabilitation.

*Model 3: With the addition of length of time of rehabilitation*.

When the relevant variables, type of surgery, TCS, MMSE, Norton, and serum urea (Table 5) were entered into the HBR model, all these variables lost statistical significance at the first step and the addition of the variable “rehabilitation time” did not affect the result, i.e., there were no independent predictors for a successful rehabilitation outcome with MRFS-R ≥ 50.

## Discussion

### Study Question 1: Is There a Difference, in Real Life Settings, Between Patients Undergoing IR and HBR?

In the HBR group, there were more married patients, fewer patients who lived alone, and more patients whose partner/spouse was their main caregiver. These findings are not very surprising. It is clear that patients who live with a functioning partner, who can take responsibility for the patient as the main caregiver, are more likely to choose HBR over IR. Thus, it would appear that the living alone is a critical factor in the decision on the rehabilitation setting. Giusti et al. (13), who evaluated the feasibility of HBR, found in a regression analysis, that living alone is the only predictor of the setting for rehabilitation (OR = 6.7).

The HBR group had more patients with intrascapular fractures and a higher rate of patients who underwent THR. These results would appear to reflect the functional and cognitive condition of HBR patients. On the one hand, patients with extracapsular fractures have longer LoS including rehabilitation and they have a higher chance of undergoing long-term care (24). On the other, THR is usually the surgery of choice if the patient was healthy prior to the fracture, with independent mobility and without cognitive impairment. Hemiarthroplasty is reserved for patients with prior mobility problems and cognitive impairment (25). Thus, the type of fracture and the subsequent choice of surgery can cause significant selection bias. This conclusion is also supported by the finding, in the present study, that the IR group had a higher rate of patients with significant chronic comorbidity (TCS), a higher number of chronic medications, and a lower Norton score.

Although the pre-surgery hemoglobin level was similar in both groups, the hemoglobin loss was more significant in the IR group. Previous studies have shown that perioperative blood loss is associated with lower mobility during physiotherapy (26), an increased risk for major adverse cardiovascular events (27), acute kidney injury (28), a longer hospital stay, and a higher rate of rehospitalization (29). Impaired kidney function tests, which were more common in the IR group, have a strong association with functional status in the future (30, 31), a greater risk of major adverse cardiovascular events (27), and even increased mortality (32).

The serum albumin level was lower in the IR group. In the vast majority of cases pre-fracture albumin tests were not available so it is difficult to conclude whether hypoalbuminemia reflects a low premorbid nutritional level or whether it is associated with perioperative stress. In any event in previous studies hypoalbuminemia was associated with postoperative complications (33, 34), a longer hospital LoS (33, 35), and even increased mortality (34, 35).

Over the course of rehabilitation more IR patients developed delirium and various infections, but had a lower rate of pressure sores. Caplan et al. (36) found that HBR, compared to IR, reduces the risk of delirium by 83%. Previous studies showed associations between hypoalbuminemia with the development of infections including surgical site infection (37), sepsis (35), and the development of delirium (14, 38). Galivanche et al. (39) found that infectious diseases, including sepsis, pneumonia, and urinary tract infections, and delirium were risk factors for the development of pressure sores. Thus, the fact that HBR patients had more pressures raises the issue of the level of daily nursing care that the patients received over the course of rehabilitation.

There were more emergency room visits in the HBR group during the 6 months following the fracture than in the IR group. We see some possible explanations: Was the comprehensive geriatric assessment by the HBR medical staff less comprehensive than we thought with insufficient attention to all the patients' medical problems? Were there medical issues that were not taken into account by the Charlson index, so the medical condition of the HBR patients was actually more complex than the score reflected?

The differences between the study groups in terms of type of surgery, TCS, rates of infectious diseases and delirium, laboratory tests, and postoperative functional decline, enabled us to answer the first study question. IR patients had fewer sources of support, more complex medical conditions, and apparently were more frail, at least as it relates to the choice of surgery.

### Study Question 2: Is There a Difference in Rehabilitation Outcomes Between the two Study Groups?

Some of the previous studies in this area reached the conclusion that HBR has advantages over IR in terms of improved function (11–13), while others did not find such a difference (10, 14, 15). The difference in results among these studies would appear to stem from differences in patient characteristics, the type of intervention, and the instruments used to measure outcome (Table 1). Although the functional condition at discharge, by FIM score, was higher in the HBR group (FIMd 91.3 ± 29.1 vs. 83.6 ± 19.0, *P* < 0.001), this results appears to reflect the fact that their admission functional condition (FIMa) was better (76.8 ± 26.9 vs. 65.1 ± 14.1, *P* < 0.001). In our opinion, the improvement in functional condition, which was significantly higher in the IR group (FIM gain 18.5 ± 13.3 vs. 14.6 ± 11.0, *P* = 0.002), does not reflect a greater level of success in the IR group. The MRFS-R score, which has advantages that were presented in a previous publication from the Geriatrics ward of the Soroka University Medical Center (23), indicates that there were no significant differences between the two study groups. Both the mean MRFS-R score and the percentage of patients with a score of MRFS-R ≥ 50 were similar (Table 3). Based on the multivariate logistic regression analysis to predict rehabilitation success (MRFS-R ≥ 50) that included all the relevant variables from the univariate analyses (Table 4) with the addition of the variable “rehabilitation setting” we found that this variable did not predict the success of rehabilitation. Thus, the results of the present retrospective study did not show any significant effect of rehabilitation setting on rehabilitation outcome.

### Study Question 3: Are There Different Predictors of Success in the two Rehabilitation Settings?

#### HBR

The univariate analyses identified predictors of rehabilitation success, defined as MRFS R ≥ 50: TCS, MMSE, Norton, and serum urea level. However, in the multivariate analysis none of these predictors or sex, type of surgery, and rehabilitation LoS were independent predictors of rehabilitation success.

#### IR

The univariate analyses identified predictors of rehabilitation success, defined as MRFS R ≥ 50: age, TCS, MMSE, Norton score, serum albumin level, delirium rate, and periprosthetic fracture. In the multivariate analysis three variables remained as independent predictors of rehabilitation success: TCS (every increase of one point reduced the chance of successful rehabilitation by 17%), MMSE (every increase of one point increased the chance of success by 9%), and serum albumin (every increase of 1 gr/dl increased the chance of success almost six-fold).

The issue of rehabilitation success has been investigated comprehensively in the past. For example, Cary et al. (40) in a study of almost 35,000 patients, following a femoral neck fracture, found that sex, age, a FIM score in the community, co-morbidity, and LoS predicted the outcome of rehabilitation, which was defined as the discharge FIM score. In a previous study conducted in the Geriatrics ward of the Soroka University Medical Center (41) was found that anamnestic FIM, age, co-morbidity (severe congestive heart failure, visual acuity impairment), MMSE score, and serum albumin level were independent predictors of rehabilitation outcome which was defined as absolute efficacy of rehabilitation and calculated as: change in FIM during rehab, divided by [126 (maximum FIM) – admission FIM] ×100.

With MRFS-R as the measure of success in another study in our department (23), we found that comorbidity, MMSE, and LoS were independent predictors of success. It should be noted that in that study serum albumin was not included in the analyses. Thus, the differences in results among the various studies, in addition to different study populations and different methods of rehabilitation, would appear to be related to the method used to measure success.

In light of the results of the present study, the answer to the question “Do the results of rehabilitation in two different frameworks have similar predictor variables?” is negative. One cannot determine the predictor variables for success in HBR while the factors that predict successful rehabilitation in IR include sex, comorbidity, and postoperative serum albumin level.

The present study has several advantages. It is a comparative study of two real-life situations among patients who underwent rehabilitation in two frameworks, both of which were affiliated with the same regional Geriatrics service whose rehabilitation staffs had an ongoing collaboration. We used computerized medical data systems that enabled us to collect a broad spectrum of data that could be introduced into a model with many relevant indices. The measure of rehabilitation success was determined in advance, using an instrument, MRFS-R, that we believe takes into account all the parameters of the patient's functional state prior to the fracture, at admission to rehabilitation, and at discharge from rehabilitation, and has the capacity of optimal adjustment for the rehabilitation potential of each specific patient (23).

The study also has several limitations. The most significant limitation is its retrospective nature and the absence of randomization. For this reason, the conclusion that there were no significant differences in rehabilitation outcomes between the two rehabilitation settings was based on data extracted from the electronic medical record. It is possible that some important information was not included. Furthermore, the results of the multivariate logistic regression did not enable us to provide a complete answer to the study question. Of course, the ultimate solution to this problem would be a randomized controlled trial.

Another significant limitation is the way the FIM questionnaires were completed. Although all the geriatricians working in the two frameworks underwent training in geriatric medicine in the Soroka University Medical Center and received a similar geriatric education, and there is a strong connection among the staffs including common staff meetings, we cannot rule out variance among the evaluators that could have affected the results of the study.

The present study has several additional limitations. Data were not collected on Instrumental ADL, so we do not know if, and to what extent, this measure changed as a result of rehabilitation in the two study populations. It is possible that when the patients are in their natural environment this measure could improve more. The absence of information on quality of life and the cost of treatment are also study limitations.

In addition, we accounted only for the number of medications that the patients were taking and not details on specific drugs. On the one hand, this information, for example, drugs for treatment of osteoporosis or sleeping pills, could add to our understanding of the causes of falls and fractures, while on the other information on drugs with anticholinergic activity could explain the differences in rehabilitation outcomes.

In this study, we evaluated the results of the intensive phase of rehabilitation only. At the end of the intensive phase the patients continued with a process of non-intensive rehabilitation in various community frameworks, in a rehabilitation center, or by once-a-week rehabilitation at home. Lack of information on the patients' functional state at the end of this rehabilitation process weakens the study conclusions and indicates the need for continuing research.

The methods used for HBR in the southern region of the Clalit Healthcare Services may be different from those used in other frameworks so, for example, every patient who is discharged to HBR receives an allowance for nursing care. In addition, the home rehabilitation staff includes a geriatrics specialist who took overall responsibility for 24/7 medical care. This is in contrast to most studies in this field where medical care was undertaken by a primary care doctor who was not an integral part of the rehabilitation staff (Table 1). Thus, caution is needed in generalizing the results of this single center study with a small number of patients to a broader population in other places.

In summary, based on the results of this retrospective study that evaluated short-term outcomes of intensive rehabilitation among patients with femoral neck fractures, patients with more complex medical conditions that underwent IR had similar rehabilitation outcomes as patients with less medically complex conditions who underwent HBR. These conclusions can serve as an additional tool for the selection of a rehabilitation framework that is tailored to the individual patient's profile.

## Data Availability Statement

The raw data supporting the conclusions of this article will be made available by the authors, without undue reservation. Requests to access these datasets should be directed to yanp@bgu.ac.il.

## Ethics Statement

The studies involving human participants were reviewed and approved by the Helsinki Committee of the Soroka Medical Center (SOR-0211-19). Written informed consent for participation was not required for this study in accordance with the national legislation and the institutional requirements.

## Author Contributions

YL, BP, and YP designed the study, collected the data, and wrote the article. EZ, DS, DK, and EM designed the study, collected the data, and assisted with writing the article. TF designed the study, was responsible for the statistical design of the study and for carrying out the statistical analysis.

## Conflict of Interest

The authors declare that the research was conducted in the absence of any commercial or financial relationships that could be construed as a potential conflict of interest.
